# Terahertz Optical Bistability in the Metal Nanoparticles-Graphene Nanodisks-Quantum Dots Hybrid Systems

**DOI:** 10.3390/nano10112173

**Published:** 2020-10-30

**Authors:** Mariam M. Tohari

**Affiliations:** Department of Physics, College of Science, King Khalid University, Abha 61413, Saudi Arabia; mrohary@kku.edu.sa

**Keywords:** terahertz optical bistability, unidirectional ring cavity, self-Kerr nonlinearity, metal-nanopaticles-graphene nanodisks-quantum dots hybrid system

## Abstract

We theoretically investigate the optical bistability in the metal nanoparticles-graphene nanodisks-quantum dots hybrid plasmonic system in the infrared regime of the electromagnetic radiation. The quantum dot is considered to be a three-level atomic-like system of Λ type interacting with probe and control fields. By using the standard model of the optical bistability where a nonlinear medium is situated in an optical ring cavity, we numerically solve the equation of motion for the density matrix elements that describe the dynamics of the system in steady-state conditions along with the boundary conditions of the cavity to analyze the optical bistability of the system. The effect of the geometrical features of the system and the parameters of the interacting fields including the strength and detuning of the fields on the optical bistability behavior are investigated. Our proposed hybrid plasmonic system shows an ultralow-threshold controllable optical bistability, providing a promising platform for optical bistable devices at the terahertz, such as all-optical switches and biosensors.

## 1. Introduction

The terahertz (THz) spectral range of the electromagnetic radiation, of the wavelength range between 1 mm to 30 μm has attracted much attention due to its wide potential applications, including sensing [1], imaging [2], spectroscopy [3], and medical diagnostic [4], because of the low attenuation of the terahertz radiation in addition to its frequencies that match the rotational and vibrational transition frequencies of molecules. Furthermore, the THz optoelectronic applications have shown rapid developments in the last decade resulting from the developments of the ultrashort laser pulses with frequency components in the THz frequency range [5]. The above applications can be enhanced by using Kerr nonlinear materials whereby the refractive index depends on the light intensity resulting from the large third-order nonlinear susceptibility of these materials [6]. By using an optical cavity filled with a resonant Kerr nonlinear material, optical bistability resulting in two transmission stable states for a certain incident state [7] has extensively been studied because of the related potential applications in the optical control including optical transistors, memory elements and all-optical switching [8,9,10,11,12,13].

The unique electronic structure of graphene resulting from the linear dispersion relation near the Dirac points provides an ultrawideband tunability as a result of Pauli blocking [14]; graphene-based materials [15,16,17] can be used as a promising platform of photonics and optoelectronics [18,19]. Moreover, it has been shown that the linear dispersion properties of graphene should result in strong nonlinear optical behavior at the microwave and THz frequencies [20,21]. Consequently, nonlinear phenomena such as optical bistability have recently been studied for several graphene-based materials in order to achieve low thresholds and efficient optical control at the THz frequencies [22,23,24,25,26]. Specifically, the optical bistability has theoretically been investigated for graphene-wrapped dielectric composite at THz frequencies. It has been shown that there is a strong dependence of both third-order nonlinear susceptibility and optical bistability on the Fermi energy of graphene, in addition to the possibility of achieving an ultralow threshold under the normal dissipation [27]. Interestingly, as a consequence of the field enhancement of graphene plasmons and large third-order nonlinear susceptibility of graphene, Guo et al. have theoretically obtained a low threshold of optical bistability of $20\;MW/cm^{-2}$, which can be further lowered to $0.5\;MW/cm^{-2}$ by using graphene nanoribbons of a one-dimensional nature [28]. Similar work has also been carried out by using a modified Kretschmann–Raetter configuration where the metal layer is replaced by the dielectric sandwich structure with the insertion of a graphene layer. It was found that the threshold intensities required to observe optical bistability are lowered due to the graphene plasmons and further lowering can be achieved by reducing the Fermi energy and increasing the thickness of the sandwich structure [22]. Moreover, an ultralow switching threshold of $1.6\;kW/cm^{-2}$ with a low Fermi energy of graphene around 2 THz has been shown analytically by using a proposed model for long-range surface plasmons resonance configuration, taking into account the advantage of the field enhancement due to the graphene plasmons [26].

Recently, giant and controllable self-Kerr nonlinearity has been demonstrated in the metal nanoparticles-graphene nanodisks-quantum dots (MNP-GND-QD) hybrid system at the optical region of the electromagnetic radiation [29]. Consequently, this novel hybrid plasmonic system has shown ultralow threshold controllable optical multistability at the optical frequencies with transitions to optical bistability under certain conditions on the geometrical features of the system and the parameters of the interacting fields [30]. However, graphene has emerged to be a promising plasmonics material for the THz applications due to the dramatic increase of graphene absorbance in the THz regime [31,32]. Moreover, at typical values of the doping level, graphene plasmons are of the THz energy [31]. Therefore, it is interesting to investigate the optical bistability of our proposed system in the THZ regime that has many important chemical, biological and optoelectronics applications.

In the present work, we theoretically investigate the THz optical bistability in the MNP-GND-QD hybrid system situated in a unidirectional ring optical cavity. The QD is modeled as a three-level atomic system interacting with probe and control fields. Due to the unique properties of graphene plasmons including tunability via doping levels and long propagation distances, GND is utilized as a resonator of the system. The MNP is used to support the plasmons of GND and provide more options for optical control. Under the boundary conditions of the unidirectional optical ring cavity, we solve the time evolution of the density matrix elements, that describe the dynamics of the confined nonlinear plasmonic system, with the propagation equation of the probe field circulating inside the cavity. The effect of the parameters of both the system setup and the interacting fields are investigated in order to achieve the ultralow threshold of the controllable optical bistability demonstrated by our proposed plasmonic system at the THz regime of the electromagnetic spectrum.

## 2. Theoretical Model

**Figure 1:**
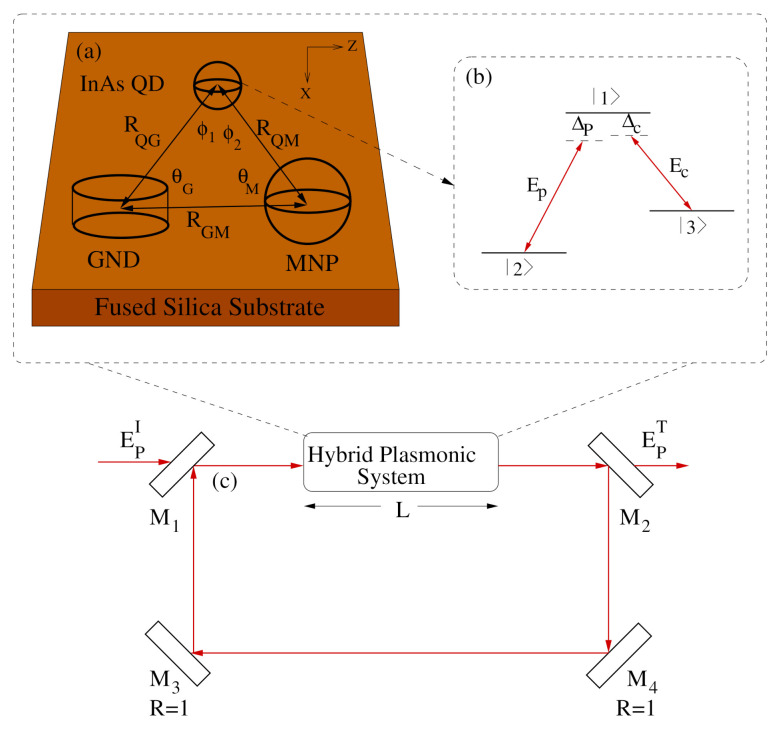
(**a**) The MNP–GND–QD hybrid system setup. (**b**) Λ-type atomic configuration of the QD. (**c**) Unidirectional ring optical cavity having the MNP–GND–QD hybrid system of length *L*. The mirrors $M_{3}$ and $M_{4}$ are perfect mirrors whereas $M_{1}$ and $M_{2}$ are partially reflecting mirrors. The incident and transmitted fields are denoted by $E_{p}^{I}$ and $E_{p}^{T}$ respectively.

We consider the MNP-GND-QD hybrid system embedded in a fused silica substrate as shown in Figure 1. The QD is considered as a Λ-type atomic configuration, where the transition |1〉↔|2〉 of the dipole moment $\mu_{12}$ is driven by the probe field $E_{p}$ of frequency $\omega_{p}$, Rabi frequency $\Omega_{p}$ and detuning $\Delta_{p}=\omega_{12}-\omega_{p}$ while the transition |1〉↔|3〉 of the dipole moment $\mu_{13}$ is mediated by the control field $E_{c}$ of frequency $\omega_{c}$, Rabi frequency $\Omega_{c}$ and detuning $\Delta_{c}=\omega_{13}-\omega_{c}$. Under the rotating wave approximation, the dipole–dipole interaction Hamiltonian resulting from the coupling between the plasmons of GND and excitons of the QD in the presence of MNP is given as [33]:

(1) $$\begin{array}{ccc} H^{RWA}= & & \hbar (\Delta_{p}\sigma_{11}+\Delta_{2}\sigma_{33})\\ & & -\hbar \left[\Omega_{p}\left(\Pi_{x}+\Phi_{x}\right)+\Lambda_{x}\rho_{12}\right]\sigma_{12}\\ & & -\hbar \left[\Omega_{c}\left(\Pi_{z}+\Phi_{z}\right)+\Lambda_{z}\rho_{13}\right]\sigma_{13}+H.C. \end{array}$$

where $\sigma_{11}$ and $\sigma_{33}$ are the projection operators onto the lower and upper levels, whereas $\sigma_{1i}$ gives the flip operators connected to the optical transitions. $\Pi_{x,z}$, $\Phi_{x,z}$ and $\Lambda_{x,z}$ are the enhancement factors due to the dipole–dipole interaction defined as [33]:

(2a) $$\begin{array}{c} \Pi_{x}=\frac{1}{4\pi \epsilon^{*}}\left[\frac{\alpha_{G}^{x}\left(3cos\phi_{1}-1\right)}{R_{QG}^{3}}+\frac{\alpha_{M}\left(3cos\phi_{2}-1\right)}{R_{QM}^{3}}\right], \end{array}$$

(2b) $$\Phi_{x}=\frac{-\alpha_{G}^{x}\alpha_{M}}{{\left(4\pi \epsilon^{*}\right)}^{2}R_{GM}^{3}}\left[\frac{3cos\phi_{1}-1}{R_{QG}^{3}}+\frac{3cos\phi_{2}-1}{R_{QM}^{3}}\right],$$

(2c) $$\Lambda_{x}=\frac{\mu_{12}^{2}}{{\left(4\pi \epsilon^{*}\right)}^{2}\hbar \epsilon_{0}\epsilon_{b}}\left[\frac{\alpha_{G}^{x}{\left(3cos\phi_{1}-1\right)}^{2}}{R_{QG}^{6}}+\frac{\alpha_{M}{\left(3cos\phi_{2}-1\right)}^{2}}{R_{QM}^{6}}\right],$$

(2d) $$\Pi_{z}=\frac{1}{4\pi \epsilon^{*}}\left[\frac{\alpha_{G}^{z}\left(3cos\theta_{G}-1\right)}{R_{QG}^{3}}+\frac{\alpha_{M}\left(3cos\theta_{M}-1\right)}{R_{QM}^{3}}\right],$$

(2e) $$\Phi_{z}=\frac{2\alpha_{G}^{z}\alpha_{M}}{{\left(4\pi \epsilon^{*}\right)}^{2}R_{GM}^{3}}\left[\frac{3cos\theta_{G}-1}{R_{QG}^{3}}+\frac{3cos\theta_{M}-1}{R_{QM}^{3}}\right],$$

(2f) $$\Lambda_{z}=\frac{\mu_{13}^{2}}{{\left(4\pi \epsilon^{*}\right)}^{2}\hbar \epsilon_{0}\epsilon_{b}}\left[\frac{\alpha_{G}^{z}{\left(3cos\theta_{G}-1\right)}^{2}}{R_{QG}^{6}}+\frac{\alpha_{M}{\left(3cos\theta_{M}-1\right)}^{2}}{R_{QM}^{6}}\right],$$

where $\alpha_{M}$ represents the polarizability of MNP given in terms of its volume and dielectric constant of the metal $\epsilon_{M}$ and $\alpha_{G}^{x}$ ($\alpha_{G}^{z}$) is the shape dependent polarizability of GND induced by x (z) polarized field [34]. $\epsilon^{*}$ is the effective dielectric constant given in terms of the dielectric constants of both the QD $\epsilon_{QD}$ and the substrate $\epsilon_{b}$. Note that the center-to-center distances $R_{QG}$, $R_{QM}$ and $R_{GM}$ are governed by the triangle law [33]. The Liouvillian term that describes the decay channels of the system is given by [33]:

(3) $$\begin{array}{ccc} L_{\rho }= & & \frac{\gamma_{13}}{2}\left(\rho \sigma_{11}+\sigma_{11}\rho -2\sigma_{31}\rho \sigma_{13}\right)\\ & & +\frac{\gamma_{12}}{2}\left(\rho \sigma_{11}+\sigma_{11}\rho -2\sigma_{21}\rho \sigma_{12}\right)\\ & & +\frac{\gamma_{32}}{2}\left(\rho \sigma_{33}+\sigma_{33}\rho -2\sigma_{23}\rho \sigma_{32}\right) \end{array}$$

where $\gamma_{1i}\left(i=2,3\right)$ is the spontaneous decay rates of QD and $\gamma_{32}$ represents the lower states dephasing. Using the above Hamiltonian and Liouvillian term, one can obtain the following time-evolution of the density matrix elements that describe the dynamics of the system [33]:

(4a) $$\begin{array}{ccc} & {\dot{\rho }}_{13}= & -\left[\left(\frac{\gamma_{13}}{2}+\frac{\gamma_{12}}{2}\right)+i\left(\Delta_{c}-\Lambda_{z}\left(\rho_{33}-\rho_{11}\right)\right)\right]\rho_{13}\\ & & +i\Omega_{c}\left(\Pi_{z}+\Phi_{z}\right)\left(\rho_{33}-\rho_{11}\right)+i\left[\Omega_{p}\left(\Pi_{x}+\Phi_{x}\right)+\Lambda_{x}\rho_{12}\right]\rho_{23}, \end{array}$$

(4b) $$\begin{array}{ccc} & {\dot{\rho }}_{12}= & -\left[\left(\frac{\gamma_{13}}{2}+\frac{\gamma_{12}}{2}\right)+i\left(\Delta_{p}-\Lambda_{x}\left(\rho_{22}-\rho_{11}\right)\right)\right]\rho_{12}\\ & & +i\Omega_{p}\left(\Pi_{x}+\Phi_{x}\right)\left(\rho_{22}-\rho_{11}\right)+i\left[\Omega_{c}\left(\Pi_{z}+\Phi_{z}\right)+\Lambda_{z}\rho_{13}\right]\rho_{32}, \end{array}$$

(4c) $$\begin{array}{ccc} & {\dot{\rho }}_{32}= & -\left(\frac{\gamma_{32}}{2}+i\Delta_{2}\right)\rho_{32}+i\left[\Omega_{c}^{*}\left(\Pi_{z}^{*}+\Phi_{z}^{*}\right)+\Lambda_{z}^{*}\rho_{31}\right]\rho_{12}\\ & & -i\left[\Omega_{p}\left(\Pi_{x}+\Phi_{x}\right)+\Lambda_{x}\rho_{12}\right]\rho_{31}, \end{array}$$

(4d) $$\begin{array}{ccc} & {\dot{\rho }}_{11}= & -\left(\gamma_{12}+\gamma_{13}\right)\rho_{11}+i\left[\Omega_{c}\left(\Pi_{z}+\Phi_{z}\right)+\Lambda_{z}\rho_{13}\right]\rho_{31}\\ & & +i\left[\Omega_{p}\left(\Pi_{x}+\Phi_{x}\right)+\Lambda_{x}\rho_{12}\right]\rho_{21}+c.c., \end{array}$$

(4e) $${\dot{\rho }}_{22}=\gamma_{12}\rho_{11}+\gamma_{32}\left(\rho_{33}-\rho_{22}\right)-i\left[\Omega_{p}\left(\Pi_{x}+\Phi_{x}\right)+\Lambda_{x}\rho_{12}\right]\rho_{21}+c.c.,$$

(4f) $${\dot{\rho }}_{33}=\gamma_{13}\rho_{11}+\gamma_{32}\left(\rho_{22}-\rho_{33}\right)-i\left[\Omega_{c}\left(\Pi_{z}+\Phi_{z}\right)+\Lambda_{z}\rho_{13}\right]\rho_{31}+c.c.,$$

where $\Delta_{2}=\Delta_{p}-\Delta_{c}$ is the two-photon detuning. Obviously, due to the dipole–dipole interaction between the components of the system, within the near field approximation, the Rabi frequencies of both the probe and control fields are enhanced by factors $|\Pi_{x}+\Phi_{x}|$ and $|\Pi_{z}+\Phi_{z}|$ respectively. Additionally, the dephasing rates of the probe and control fields are also enhanced by $\Lambda_{x}$ and $\Lambda_{z}$ respectively.

We consider the MNP-GND-QD hybrid system in a unidirectional ring cavity, as shown in Figure 1c. To simplify, the mirrors $M_{3}$ and $M_{4}$ are considered to be perfect reflectors. The partially transmitting mirrors $M_{1}$ and $M_{2}$ are used to analyze the optical bistability by measuring the input and output beams. The probe field that circulates in the optical cavity inducing the atomic polarization $P\left(\omega_{p}\right)=N\mu_{21}\rho_{21}$, where *N* is the atomic number density of the QD, is governed by Maxwell’s equation under the slowly varying envelop approximation [6]:

(5) $$\frac{\partial E_{p}}{\partial t}+c\frac{\partial E_{p}}{\partial z}=i\frac{\omega_{p}P\left(\omega_{p}\right)}{2\epsilon_{0}}$$

By solving the propagation equation of the probe field with the boundary conditions of a perfect tuned cavity of length *L* in the steady-state limit [7]:

(6a) $$\begin{array}{c} E_{p}\left(L\right)=\frac{E_{p}^{T}}{\sqrt{T}} \end{array}$$

(6b) $$E_{p}\left(0\right)=\sqrt{T}E_{p}^{I}+RE_{p}^{T}\left(L\right)$$

one can obtain the following normalized input-output relationship in the dimensionless form:

(7) $$Y=X-iC\rho_{21}$$

where $Y=\mu_{12}E_{p}^{I}/\hbar \gamma_{12}\sqrt{T}$ and $X=\mu_{12}E_{p}^{T}/\hbar \gamma_{12}\sqrt{T}$. Note that the second term of Equation (7) is very important in achieving the optical bistability because it is given in terms of the feedback provided by the optical cavity and the inserted Kerr nonlinear plasmonic medium, where $C=LN\omega_{p}\mu_{12}^{2}/2Tc\epsilon_{0}\hbar \gamma_{12}$ is the dimensionless usual cooperation parameter.

## 3. Results and Discussion

In order to investigate the THz optical bistability in the MNP-GND-QD hybrid system, we consider a monolayer of GND of the thickness $L_{x}=0.35\;nm$ and the radius of $L_{z}=7\;nm$ [35]. The dielectric constant of GND is calculated by using $\epsilon_{\infty }=1.964$, plasma energy of $\hbar \omega_{p}=6.02\;eV$ and damping rate of $\gamma_{G}=5\;T\mathrm{Hz}$ corresponding to the carrier mobility of $\mu =10^{4}\;cm^{2}V^{-2}s^{-1}$ and the Fermi energy of 0.2 eV [36,37,38]. For the GND of these parameters deposited on the fused silica substrate of $\epsilon_{b}=2.081$, the surface plasmon resonances are; $\hbar \omega_{sp}^{x}=0.8026\;eV$ and $\hbar \omega_{sp}^{z}=4.1250\;eV$. In order to examine the THz optical bistability in our proposed system, we adopt the plasmon energy of $\hbar \omega_{sp}^{x}$ for the GND. Consequently, the probe field (control field) is applied along the x-axis (z-axis). To support the plasmons of GND, we use a spherical silver nanoparticle of $\epsilon_{\infty }=5.7$, plasma frequency of $\omega_{p}=1.36\times 10^{16}\;s^{-1}$, and damping rate of $\gamma_{M}=10^{14}\;s^{-1}$. The QD is chosen to be InAs QD that has an energy transition resonant with the energy of GND plasmons, dielectric constant of $\epsilon_{QD}=12$, damping width of $\Gamma_{21}=1.137\;\mu eV$ and dipole moments of $\mu_{12}=\mu_{13}=0.1\;e\;nm$ where $\mu_{13}$ and $\mu_{12}$ in the InAs QD are aligned perpendicular to each other [39].

In the following, we discuss the controlling of the optical bistability in the MNP-GND-QD hybrid system via the parameters of the system setup, including the size of MNP and the edge-to-edge distances between the GND and the MNP, in addition to the detuning and strength of both the probe and control fields in order to optimize the threshold of controllable optical bistability in the proposed MNP-GND-QD hybrid plasmonic system. Qualitatively, the input-output relationship is in good agreement with that experimentally found by Joshi et al. [40], using the same standard model.

**Figure 2:**
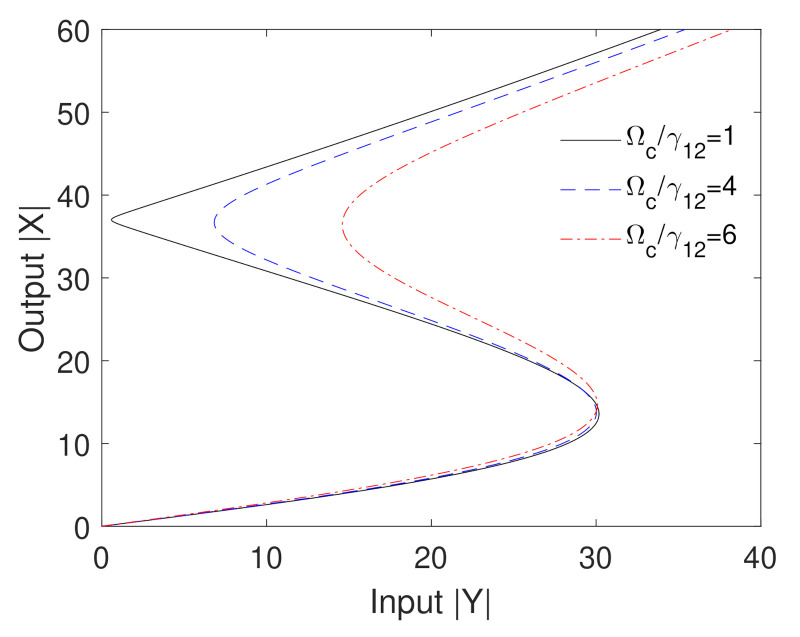
Output versus input for different values of the Rabi frequency of the control field. The other parameters are $\frac{\Delta_{p}}{\gamma_{12}}=0$, $\frac{\Delta_{c}}{\gamma_{12}}=6$, $R_{M}=20\;nm$, R=5 nm, $\theta_{M}=0.5\;\mathrm{rad}$, $\theta_{G}=1\;\mathrm{rad}$, C=195.34 and the mobility of GND is $\mu =10^{4}\;cm^{2}V^{-2}s^{-1}$.

The effect of the Rabi frequency of the control field on the optical bistability behavior is examined in Figure 2. It can be seen that the thresholds of the optical bistability is not sensitive to the Rabi frequency of the control field. However, the decreasing of $\Omega_{c}$ leads to a significant broadening of the hysteresis loop of the optical bistability. This can be attributed to the role of the control field in the reduction of the absorption of the probe field leading to a decrease in the self-Kerr nonlinearity and a narrowing of the resulting hysteresis loop of the bistability. Thus, the optical bistability behavior can be externally controlled via the strength of the control field, which can help to construct efficient optical bistable devices.

**Figure 3:**
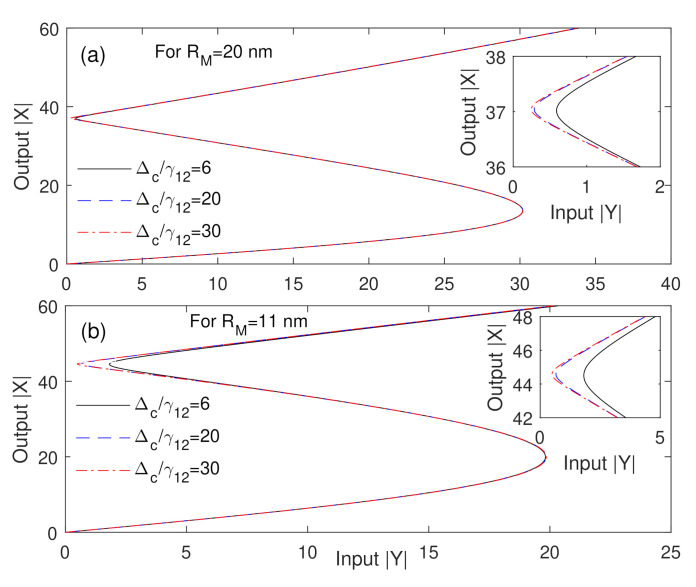
Output versus input for different values of the detuning of the control field at two values of the MNP radius; (**a**): $R_{M}$ = 20nm and (**b**): $R_{M}$ = 11nm. The other parameters are $\frac{\Delta_{p}}{\gamma_{12}}=0$, $\frac{\Omega_{c}}{\gamma_{12}}=1$, R=5nm, $\theta_{M}=0.5\;\mathrm{rad}$, $\theta_{G}=1\;\mathrm{rad}$, C=195.34 and the mobility of GND is $\mu =10^{4}\;cm^{2}V^{-2}s^{-1}$.

Additionally, we explore in Figure 3 the influence of the detuning of the control field on the behavior of optical bistability for two values of the MNP radius. Remarkably, the threshold of the optical bistability is not affected by the detuning of the control field. However, the sensitivity of the width of the hysteresis loop of the optical bistability to the detuning of the control field clearly emerges for the relatively small size of MNP, as shown in Figure 3b. Moreover, the sensitivity of the optical bistability behavior of the MNP-GND-QD hybrid system of the small size of MNP is missing for the detuning of the control field larger than $20\gamma_{12}$, as shown in Figure 3b. We can interpret this result in terms of the enhancement factors due to the dipole-dipole interaction, that are strongly affected by the size of MNP. Specifically, for a relatively small MNP, the polarizability of MNP and GND induced by the probe field will be comparable, leading to the higher sensitivity of the system. This result provides another advantage of our proposed hybrid plasmonic system that paves the way to controllable optical bistable devices.

**Figure 4:**
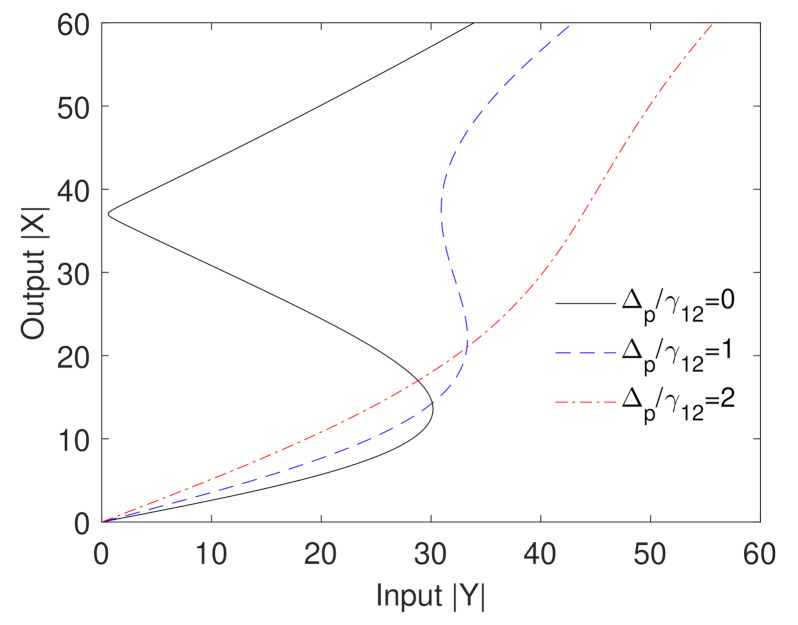
Output versus input for different values of the detuning of the probe field. The other parameters are $\frac{\Delta_{c}}{\gamma_{12}}=6$, $\frac{\Omega_{c}}{\gamma_{12}}=1$, $R_{M}=20\;nm$, R=5nm, $\theta_{M}=0.5\;\mathrm{rad}$, $\theta_{G}=1\;\mathrm{rad}$, C=195.34 and the mobility of GND is $\mu =10^{4}\;cm^{2}V^{-2}s^{-1}$.

The effect of the probe field detuning on the optical bistability behavior is examined in Figure 4. It can be seen that the optical bistability is highly sensitive to the detuning of the probe field. Specifically, the ultralow threshold optical bistability is obtained when the probe field that induces the plasmons of GND is resonant with excitons in the QD due to the ultrafast energy transfer in such a case. Obviously, the threshold of optical bistability increases and the width of the hysteresis loop decreases as the detuning of the probe field increases. When the detuning of the probe field doubles the decay rate induced by the probe field, the optical bistability behavior is missed. Interestingly, the high sensitivity of the optical bistability to the probe field detuning can be employed to implement all-optical transistors, switches and optical memories.

**Figure 5:**
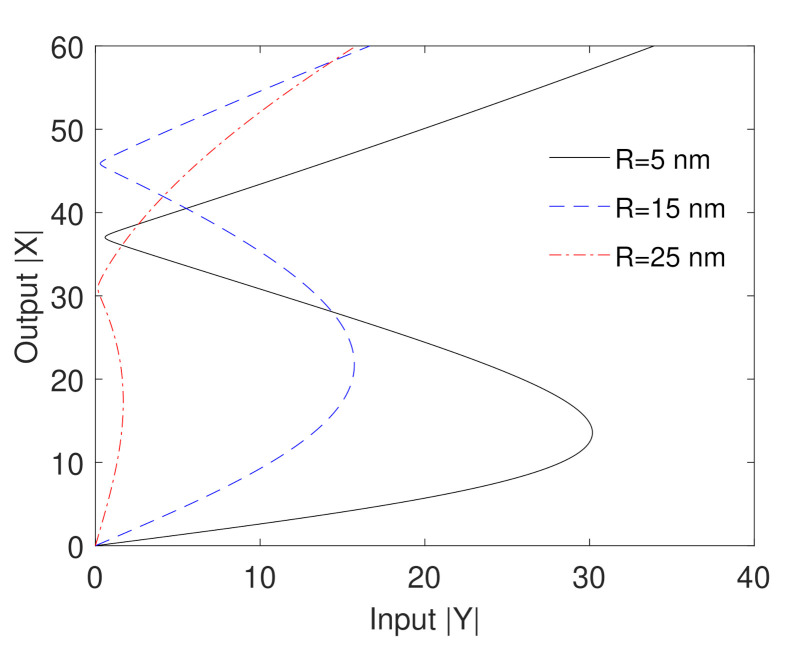
Output versus input for different values of the edge-to-edge distances between MNP and GND. The other parameters are $\frac{\Delta_{c}}{\gamma_{12}}=6$, $\frac{\Delta_{p}}{\gamma_{12}}=0$, $\frac{\Omega_{c}}{\gamma_{12}}=1$, $\theta_{M}=0.5\;\mathrm{rad}$, $\theta_{G}=1\;\mathrm{rad}$, $R_{M}=20\;nm$, C=195.34 and the mobility of GND is $\mu =10^{4}\;cm^{2}V^{-2}s^{-1}$.

Because the dynamics of our proposed plasmonic system described by Equation (4) is derived by using the dipole-dipole interaction Hamiltonian that is sensitive to the distances between the components of the system, we discuss in Figure 5 to what extent we can control the optical bistability behavior by the edge-to-edge distances between MNP and GND (R). Note that the center-to-center distance between MNP and GND ($R_{MG}$) is given in terms of (R) as: $R_{MG}=R_{M}+L_{z}+R$, where $R_{M}$ is the radius of the MNP. It can be seen that the optical bistability behavior is strongly affected by the edge-to-edge distances between MNP and GND. Specifically, ultralow threshold of bistability is obtained for the moderate value of the edge-to-edge distances between MNP and GND (R=15) i.e., Y=16. For the typical values of $\gamma_{12}$ and the transmission *T* ($\gamma_{12}=2\times 10^{9}\;s^{-1}$ and T=0.03), the value of Y=16 corresponds to the probe incident field of strength $E_{p}^{I}=36.5\;kVm^{-1}$ and intensity of $2.13\;kW{\mathrm{cm}}^{-2}$. Moreover, the width of the hysteresis loop decreases as the edge-to-edge distances between MNP and GND increase until the optical bistability behavior disappears, as shown in Figure 5 for R=25nm due to the weakness of the dipole-dipole interaction at large distances between the components of the system. Note that the giant self-Kerr nonlinearity of our proposed system that has been shown in Ref [29] is obtained for the QD near the MNP and GND due to the field enhancement of these plasmonic components that enhance the nonlinearity of the QD via the dipole–dipole interaction.

**Figure 6:**
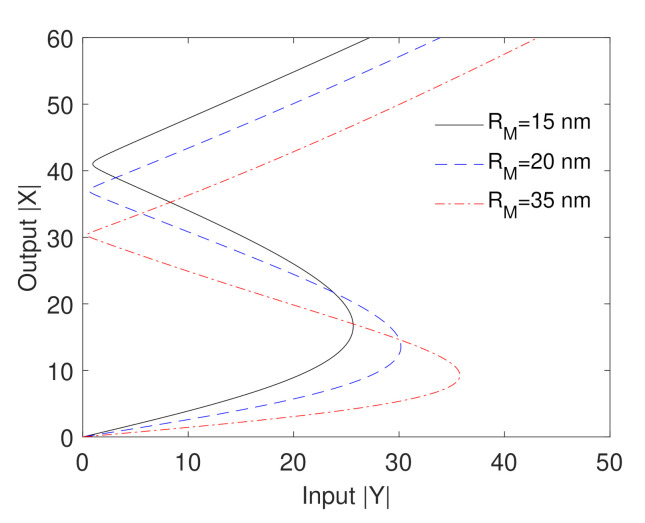
Output versus input for different sizes of the MNP. The other parameters are $\frac{\Delta_{c}}{\gamma_{12}}=6$, $\frac{\Delta_{p}}{\gamma_{12}}=0$, $\frac{\Omega_{c}}{\gamma_{12}}=1$, $\theta_{M}=0.5\;\mathrm{rad}$, $\theta_{G}=1\;\mathrm{rad}$, R=5nm, C=195.34 and the mobility of GND is $\mu =10^{4}\;cm^{2}V^{-2}s^{-1}$.

The impact of the MNP size on the optical bistability behavior is examined in Figure 6. We observe that the width of the hysteresis loop and the threshold of the optical bistability increase as the size of the MNP increases. This result can be interpreted in terms of the polarizability of the MNP which increases when increasing the size of the MNP leading to large values of the enhancement factors given by Equation (2), due to the strong dipole–dipole interaction in such cases. Therefore, the sensitivity of the optical bistability to the size of the MNP can provide another option of the optical control of bistable devices that can be constructed using our proposed plasmonic system.

To conclude, compared to the relevant studies of the optical bistability behavior in the THz for simpler systems such as graphene-coated cylindrical core-shell nanoparticles [24], graphene in thin layers of dielectrics [25] and graphene sheets [26], our proposed plasmonics system shows ultralow thresholds of the controllable optical bistability even though we have used small Fermi energy compared to those used in the indicated studies. Interestingly, it is expected that increasing the Fermi energy will enhance the nonlinearity of the system due to the large plasma frequency of graphene in such cases. However, our proposed plasmonic system consists of QD to compensate for the plasmonic losses which must be resonant with the GND plasmons. The energy of the latter can be controlled by the Fermi energy and the size of the GND, as well as the dielectric constant of the background [41]. Thus, we can-not dependently change the Fermi energy without accounting for the change of the GND plasmonic energy accordingly, which requires a suitable choice of the QD to achieve the resonant energy transfer between the plasmons of the GND and excitons of the QD. Therefore, future work may include the investigation of the optical bistability in the MNP-GND-QD hybrid system at the THz with higher Fermi energy in the GND and suitable THz QDs of resonant transition energy.

## 4. Conclusions

We have studied the optical bistability behavior in the MNP-GND-QD hybrid plasmonic system at the THz frequencies. We have found that the width of the hysteresis loop and the thresholds of the optical bistability can be controlled by the system setup in addition to the detuning and strength of both the probe and control fields. The optical bistability of our proposed system shows a high sensitivity to the detuning of the probe field and the center-to-center distances between the components of the system. Moreover, our plasmonic system has demonstrated ultralow optical bistability thresholds, implying that the proposed MNP-GND-QD hybrid plasmonic system can be employed to construct efficient controllable optical bistable devices that are also used as all-optical switches and optical transistors as well as medical biosensors. We hope that our study will stimulate future experimental investigations of the optical bistability in the MNP-GND-QD hybrid system which contributes a better understanding of this novel system and its nonlinear potential applications.
